# Supplementary material for: Plasmalogens and their Associations with Brain Function and Structure in Older Community Dwelling African Americans
Source: medRxiv. 2026 Mar 27:2026.03.26.26349429. Preprint. [Version 1] doi: 10.64898/2026.03.26.26349429 (PMC13042131; doi:10.64898/2026.03.26.26349429)
Supplement: Supplement 1 [file NIHPP2026.03.26.26349429v1-supplement-1.pdf]

medRxiv preprint doi: https://doi.org/10.64898/2026.03.26.26349429; this version posted March 27, 2026. The copyright holder for this preprint
(which was not certified by peer review) is the author/funder, who has granted medRxiv a license to display the preprint in perpetuity.
It is made available under a CC-BY 4.0 International license .

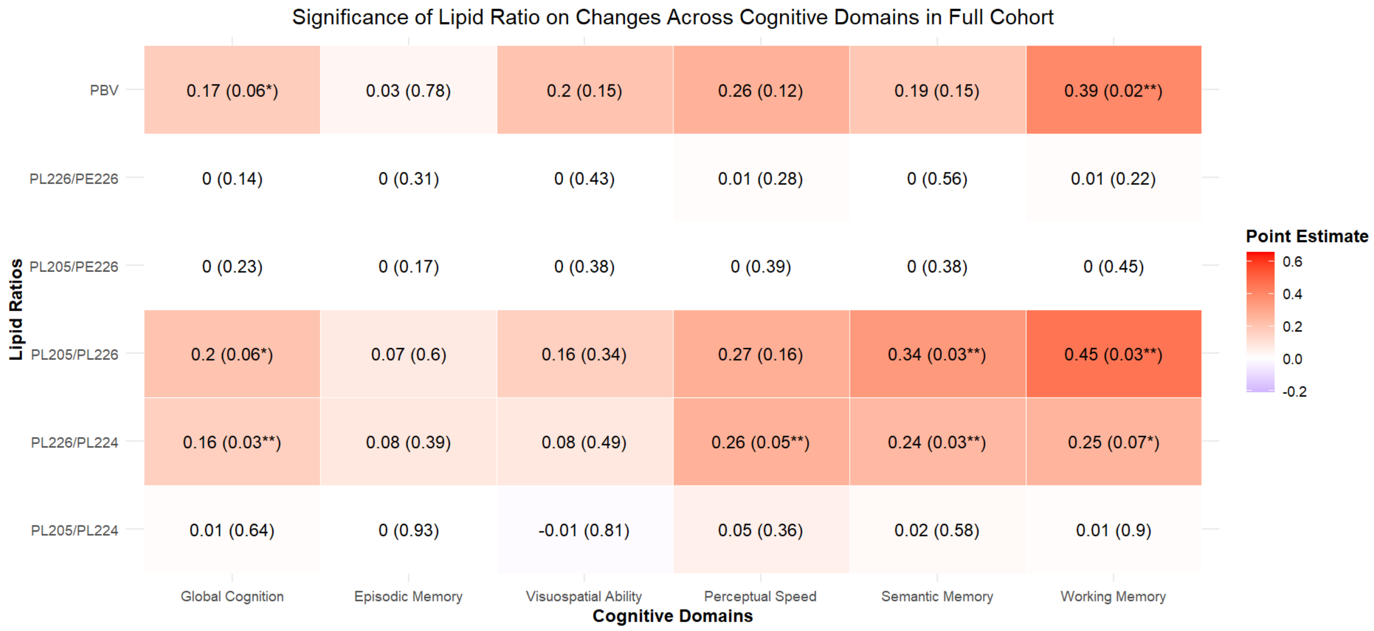

Supplementary Figure 1. Heat map of point estimates of linear regression models for all participants with cognitive testing available. Labeled as estimate(p-value) with p-values labeled p≤0.05 "**", and <0.10 "*" for clarity. Abbreviations: PBV = Plasmalogen Biosynthesis Value.

medRxiv preprint doi: https://doi.org/10.64898/2026.03.26.26349429; this version posted March 27, 2026. The copyright holder for this preprint (which was not certified by peer review) is the author/funder, who has granted medRxiv a license to display the preprint in perpetuity.
It is made available under a CC-BY 4.0 International license .

Supplementary Table 1. Linear Regression Models of Lipidomic Ratios on Cognitive Scoring in Women and Men with MRI available (n=254). Model 1 includes PBV with other covariates; Model 2 includes WMH with covariates; Model 3 includes PBV and WMH along with covariates; Model 4 includes PBV, WMH, and the PBV*WMH interaction along with same covariates. P values are labeled p≤0.05 "**", and ≤0.10 "*" for clarity. Abbreviations: PBV = Plasmalogen Biosynthesis Value; BMI = Body Mass Index; WMH = White Matter Hyperintensity.

**A) Full MRI Subset : Global Cognition**				
	**Model 1**	**Model 2**	**Model 3**	**Model 4**
**PBV**	0.13 (0.09, 0.17)	N/A	0.10 (0.09, 0.29)	0.08 (0.12, 0.48)
**WMH**	N/A	-0.14 (0.05, 0.01) **	-0.13 (0.06, 0.02)**	-0.14 (0.09, 0.10)*
**Age**	-0.01 (0.00, 0.01)**	-0.01 (0.00, 0.05)**	-0.01 (0.00, 0.07)*	-0.01 (0.00, 0.07)*
**Education**	0.05 (0.01, 0.00)**	0.05 (0.01, 0.00)**	0.05 (0.01, 0.00)**	0.05 (0.01, 0.00)**
**Total Cholesterol**	0.00 (0.00, 0.05)**	0.00 (0.00, 0.04)**	0.00 (0.00, 0.03)**	0.00 (0.00, 0.04)**
**BMI**	0.00 (0.00, 0.32)	0.00 (0.00, 0.40)	0.00 (0.00, 0.36)	0.00 (0.00, 0.35)
**PBV*WMH**	N/A	N/A	N/A	-0.04 (0.19, 0.84)
**B) Women : Global Cognition**				
	**Model 1**	**Model 2**	**Model 3**	**Model 4**
**PBV**	0.24 (0.11, 0.03) **	N/A	0.22 (0.11, 0.05)**	0.20 (0.14, 0.16)
**WMH**	N/A	-0.14 (0.06, 0.02) *	-0.13 (0.06, 0.04)*	-0.16 (0.11, 0.15)
**Age**	-0.01 (0.00, 0.03)**	-0.01 (0.01, 0.05)**	-0.01 (0.01, 0.09)*	-0.01 (0.01, 0.10)*
**Education**	0.05 (0.01, 0.00)**	0.05 (0.01, 0.00)**	0.05 (0.01, 0.00)**	0.05 (0.01, 0.00)**
**Total Cholesterol**	0.00 (0.00, 0.03)**	0.00 (0.00, 0.04)**	0.00 (0.00, 0.02)**	0.00 (0.00, 0.02)**
**BMI**	0.01 (0.01, 0.28)	0.00 (0.01, 0.46)	0.00 (0.01, 0.34)	0.01 (0.01, 0.33)
**PBV*WMH**	N/A	N/A	N/A	-0.07 (0.23, 0.76)
**C) Men : Global Cognition**				
	**Model 1**	**Model 2**	**Model 3**	**Model 4**
**PBV**	0.08 (0.21, 0.70)	N/A	0.01 (0.22, 0.97)	-0.11 (0.29, 0.70)
**WMH**	N/A	-0.19 (0.14, 0.18)	-0.19 (0.15, 0.20)	-0.23 (0.16, 0.16)
**Age**	-0.01 (0.01, 0.28)	0.00 (0.01, 0.77)	0.00 (0.01, 0.77)	0.00 (0.01, 0.76)
**Education**	0.05 (0.02, 0.02)**	0.05 (0.02, 0.00)**	0.05 (0.02, 0.03)**	0.04 (0.02, 0.06)*
**Total Cholesterol**	0.00 (0.00, 0.36)	0.00 (0.00, 0.39)	0.00 (0.00, 0.40)	0.00 (0.00, 0.41)
**BMI**	0.00 (0.01, 0.99)	0.00 (0.01, 0.89)	0.00 (0.01, 0.90)	0.00 (0.01, 0.91)
**PBV*WMH**	N/A	N/A	N/A	-0.33 (0.52, 0.53)
